# Properties of Laser-Welded Zr-Al-Co-Nb Bulk Metallic Glass

**DOI:** 10.3390/ma19061078

**Published:** 2026-03-11

**Authors:** Huei-Sen Wang, Chih-Chun Hsieh, Hou-Guang Chen, Shao-Chi Wu, Jason Shian-Ching Jang, Kuo-Jung Lee

**Affiliations:** 1 Department of Materials Science and Engineering, I-Shou University, Kaohsiung City 84001, Taiwan; huei@isu.edu.tw (H.-S.W.); houguang@isu.edu.tw (H.-G.C.); 99rws305@gmail.com (S.-C.W.); krlee@isu.edu.tw (K.-J.L.); 2 Institute of Material Science and Engineering, National Central University, Taoyuan 32001, Taiwan; jscjang@ncu.edu.tw

**Keywords:** welding, bulk metallic glass, microstructure, microhardness, thermal properties, corrosion resistance

## Abstract

In this study, the Nd:YAG laser process was employed with preselected welding parameters and varying initial welding temperatures (including room temperature, 10 °C, and 0 °C) for spot welding of (Zr_53_Al_17_Co_29_)Nb_1_ bulk metallic glass. Following welding, the microstructure—including the parent material, heat-affected zone (HAZ), and weld fusion zone (WFZ)—as well as the microhardness, thermal properties, and corrosion resistance of the welds, were systematically investigated. Owing to the low glass-forming ability of the alloy, a small amount of Zr_6_CoAl_2_ phase was observed within the amorphous matrix at the center of the bulk metallic glass cast plate. After the laser welding, sub-micron or nanoscale Zr(Al_x_Co_1−x_)_2_ phases have formed in the HAZ of all welded samples, which significantly influenced the microhardness, thermal properties, and corrosion resistance in this region. As the initial welding temperature decreased, both the volume fraction and the density of the Zr(Al_x_Co_1−x_)_2_ phase were reduced. Notably, for the weld performed at the lowest initial temperature of 0 °C, small crystalline phases were detected only at approximately 70 μm below the surface of the HAZ. To clarify the effect of IWTs on corrosion resistance, welded samples were immersed in 6 M HCl at 35 °C for 72–120 h. Surface morphologies after corrosion were examined by SEM in the PM, HAZ, and WFZ. No evident pitting was detected after 72 h of immersion. After 120 h, pitting corrosion was observed on the HAZ surfaces of welds subjected to RT and 10 °C IWTs, whereas no obvious pitting was found at an IWT of 0 °C. The pit size and density in the HAZ increased with increasing IWT. In contrast, no pitting was observed in the WFZ under any IWT condition.

## 1. Introduction

Under the consideration of biocompatibility, a Zr-Al-Co bulk metallic glass (BMG) alloy, which is free from toxic elements Cu and Ni, has been developed for biomedical applications [1,2]. Like the traditional Zr-Cu-based BMG, the Zr-Al-Co BMG [3,4,5,6,7,8,9,10] possesses several superior properties, such as high strength, hardness, wear resistance, and excellent corrosion resistance. From the thermodynamic perspective, a previous study [1] indicated that an alloy with the composition of Zr_54_Al_17_Co_29_ exhibits excellent thermal stability and can be cast into a rod with a maximum diameter of 6 mm. In terms of toughness, ductility, and plastic deformation, the alloy system has relatively poor performance. To improve its toughness, previous studies have attempted to add a fourth and softer element, Ta [1,11] or Nb [10,12,13], to improve the toughness and ductility of the BMG alloy. Regarding the Nb element, Guan et al. [14] reported that the addition of Nb improves the corrosion resistance, mechanical properties, and bioactivity of the Zr-Al-Co BMG system.

However, Matthews et al. [11] found that the glass forming ability (GFA) value of the Zr-Al-Co BMG system is relatively low when compared to that of the traditional Zr-Cu based BMG [15,16,17,18], such as Zr-Cu-Ag-Al or Zr-Cu-Ni-Al BMG systems. Moreover, when a fourth element, such as Nb [10,12,13], is added to the Zr-Al-Co BMG, it is expected that the initial low GFA may be affected, which poses a major challenge to the weldability of the Zr-Al-Co-Nb BMG alloy systems. To broaden applications of the BMG system, many welding techniques have been employed to produce a robust weld for the BMG alloy. Among these welding processes, laser welding [11,19,20,21,22] is widely used due to its advantages of lower power energy and high power density. Laser welding tends to produce the desired heating and cooling cycles within a range that does not introduce additional precipitates into the heat-affected zone (HAZ) or weld fusion zone (WFZ) of the welds. However, in some cases HAZ crystallization is still unavoidable. Previous studies have suggested that the use of the laser welding process, plus a liquid cooling device (LCD) [11,23], might lower the initial welding temperature (IWT), thereby reducing the heating and cooling time of the welding thermal cycles, and further decreasing the formation of precipitates in the welds.

To date, the effects of additional Nb in (Zr_54-x_Al_17_Co_29_) Nb_x_ on the weldability of the BMG have not been reported. Furthermore, Yu et al. [13] suggested that among the various designed Nb additions, minor-alloying of 1.0 at.% Nb into the Zr-Al-Co BMG system may provide optimal thermal stability for the GFA of the alloy system, which can be cast into a rod with a maximum diameter of 4 mm. Therefore, (Zr_53_Al_17_Co_29_) Nb_1_ was employed for Nd:YAG laser spot welding with an LCD in this study. The laser pulse shape was determined using an empirical method that focuses on weld morphology and complete penetration of a 1 mm (Zr_53_Al_17_Co_29_) Nb_1_ plate. After the welding process, the microstructure (including parent material (PM), HAZ, and WFZ), microhardness, thermal properties, and corrosion resistance of the welds were investigated.

## 2. Experimental Procedures

Ingots with a normal composition of (Zr_53_Al_17_Co_29_) Nb_1_ were prepared by arc melting mixtures of Zr, Al, Co, and Nb (the purities of the elements ≥ 99.9%) in a Ti-gattered high-purity argon atmosphere. All the ingots were re-melted more than twice to achieve chemical homogeneity. After the re-melting was completed, the BMG was formed into a 2 mm thick cast plate using the copper mold suction casting technique. The cast was then machined to a 1 mm thick, 20 mm wide, and 20 mm long plate. The cast plate was ground and polished with the 2000 grit silicon carbide paper to eliminate the surface dirt. The BMG plate was then initially characterized using X-ray diffractometry (XRD, Scintag X-400, Thermo Fisher Scientific, Santa Clara, CA, USA), a scanning electron microscope (SEM; Hitachi S-4700, Hitachinaka, Japan) equipped with an energy dispersive spectrometer (EDS; HORIBA 7200-H, Tokyo, Japan), and a differential scanning calorimetry (DSC; Netzsch 404C, NETZSCH-Gerätebau GmbH, Selb, Germany) at a heating rate of 20 K/min. The initial parameters for the laser spot welding are shown in Table 1, which are based on previous studies [11,19,24] and empirical results. The table shows the laser energy required to penetrate the 1 mm thick BMG plate. After the initial welding, this study referred to the morphologies and defect conditions of the welds, and the optimal laser welding parameters were selected. The selected parameters were then applied at progressively lower IWTs, from room temperature to 10 °C to 0 °C, using a liquid cooling device (LCD). The details of the welding processes in combination with the LCD can be reviewed in previous studies [11,23]. The microstructure of the welded BMG (including HAZ and WFZ) was investigated by optical microscopy (OM, Olympus BX51M. Olympus Corporation, Tokyo, Japan) and SEM with EDS. The detailed microstructures of the welds were investigated and compared using transmission electron microscopy (TEM; JEM-2100F/200 KV, JEOL Ltd., Tokyo, Japan). To obtain the TEM foils from specific sites, this study focused on an ion beam (FIB, SMI3050, Hitachi High-Tech, Oyama-cho, Japan) with 5 keV. Then, the thermal behaviors (the characteristic temperatures of the BMG, including glass transition temperature, T_g_; crystallization temperature, T_x_; and liquidus temperature, T_l_) of the welds, were investigated using DSC. The hardness of each area of the welds was examined using a micro-hardness tester under a load of 300 g and a loading time of 10 s.

To understand the corrosion behavior of (Zr_54_Al_17_Co_29_)_100−x_Nb_x_ (x = 0.5 wt% or 1.0 wt%), the corrosion resistance of the BMGs was evaluated in accordance with the ASTM G31-21 standard [25]. The specimens were immersed in a 6 M hydrochloric acid (HCl) solution and maintained in a thermostatically controlled water bath at 35 °C for different immersion periods ranging from 72 to 120 h, following the procedures described in the literature [26,27]. After immersion, the specimens were removed from the test bath and rinsed with 99.5% ethanol to eliminate residual solution. The surface morphologies and pitting corrosion features of the PM, HAZ, and WFZ were subsequently examined using SEM.

## 3. Results and Discussion

**Figure 1:**
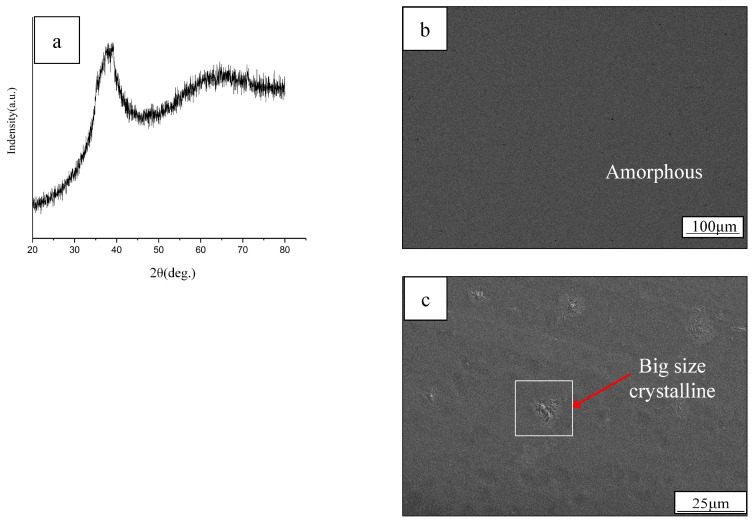
(**a**) XRD pattern (scanning on the surface of the plate) and SEM images on (**b**) the surface and (**c**) the cross-section of the plate of the BMG plate.

Figure 1a–c show the XRD pattern (scanning on the surface of the plate) and the SEM images (including the surface and cross-section of the plate) of the as-cast plate of (Zr_53_Al_17_Co_29_) Nb_1_ alloy. The XRD result, obtained from the BMG plate surface, shows no obvious crystalline peak, except for a broad halo diffraction pattern in the range of 30–50°, which indicates its glassy nature. Nevertheless, a small amount of micro-size crystalline was observed at the depth of approximately 60 μm below the surface of the BMG cast plate. EDS analysis showed that the micro-sized crystalline structure has rich Zr/Al/Co elements. More details of the micro-sized crystalline structures were identified by TEM and are discussed in a later section.

**Figure 2:**
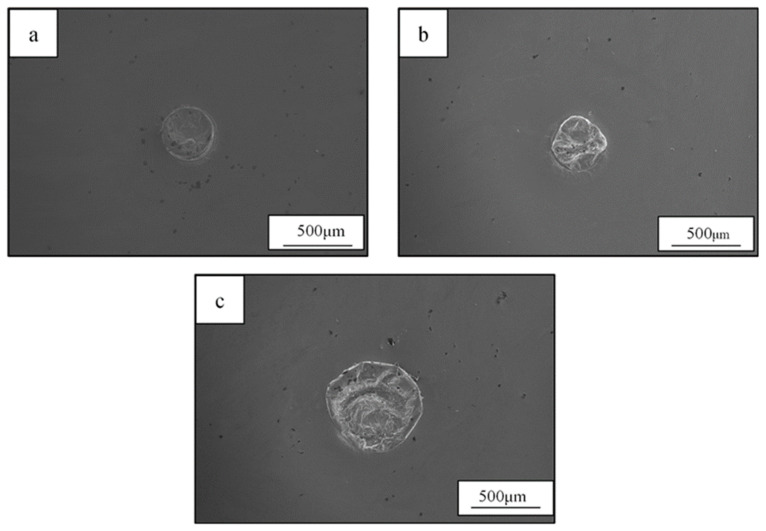
The penetration conditions of the welded plates observed from the bottom of the welded plate at welding parameters: (**a**) Condition A, (**b**) Condition B, and (**c**) Condition C.

**Figure 3:**
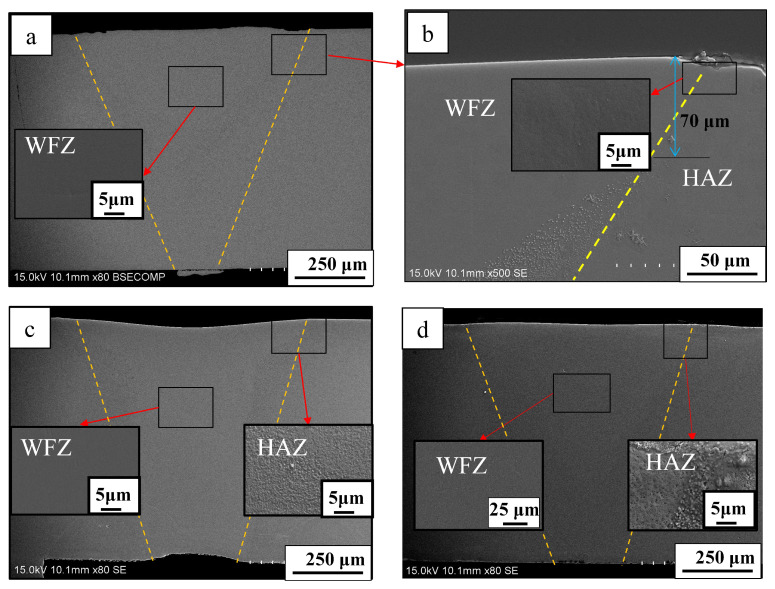
Cross-section observations from IWT of (**a**) 0 °C, (**b**) the higher magnification image at the top surface area of (**a**), (**c**) 10 °C (**d**) room-temperature (RT) welds. Note: Dashed lines represent the boundaries between regions.

**Figure 4:**
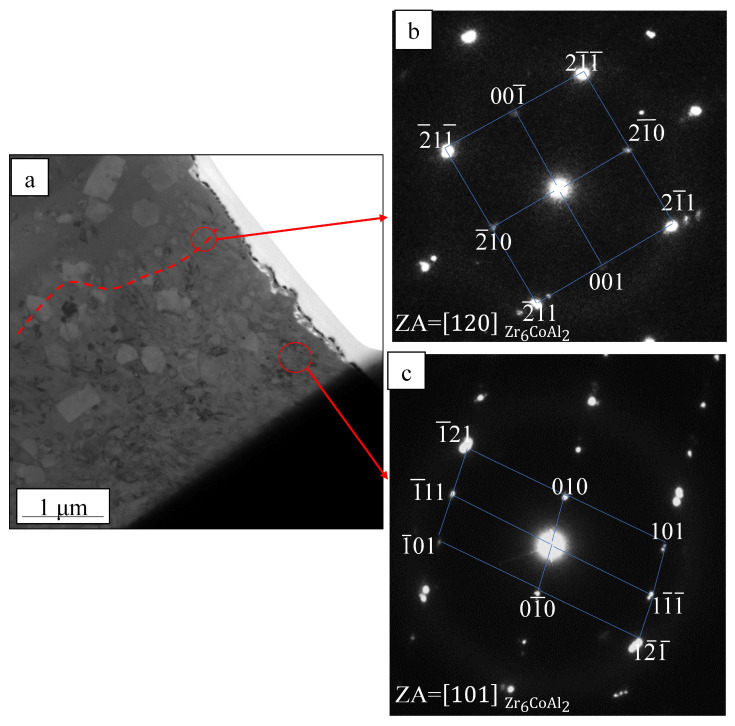
(**a**) Bright-field (BF) image of the interface between the micro-size crystalline and BMG; the corresponding selected area electron diffraction patterns, taken from the various regions of the micro-sized crystalline structure, which were identified as the Zr_6_CoAl_2_ phase, viewed along (**b**) [120] and (**c**) [101] zone axis, respectively.

**Figure 5:**
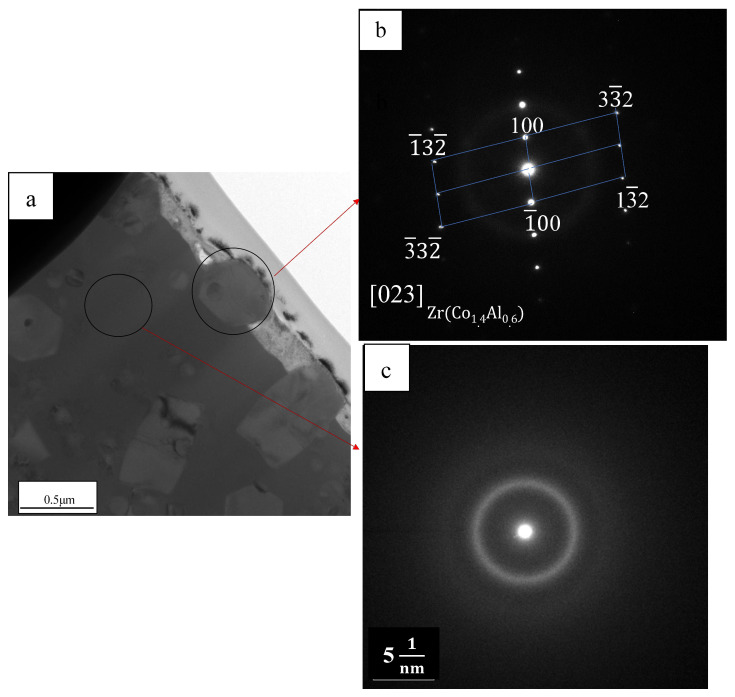
(**a**) BF of a small (sub-micro or nano-) size crystalline in the HAZ (**b**) and SAED patterns taken from small-size crystalline in the HAZ were identified as Zr(Al_x_Co_1−x_)_2_ phase viewed along [023] zone axis. (**c**) SAED patterns taken from the matrix in the HAZ.

Figure 2 shows the penetration conditions of the spot-welded BMG plates, as observed from the bottom of the welded plate. As shown in Figure 2, all selected welding parameters penetrated the 1 mm thick plate and no obvious surface defects were observed. Furthermore, as the laser energy increased, the penetration condition became more obvious. To avoid the incomplete penetration of the welded BMG plate, as caused by the low IWT, the higher laser energy parameters (i.e., welding Condition C) were selected. Using welding condition C, the BMG was spot welded with progressively lower IWTs, from room temperature 10 °C to 0 °C, using the LCD. All the weld samples were fully penetrated, as shown in Figure 3. While no obvious crystalline phases or structures were found in the WFZs of the welds, the HAZ crystallization of all the samples became unavoidable, even when laser welded at the lower IWT of 0 °C. However, for the IWT of 0 °C weld, small-sized crystalline particles were found at 70 μm below the surface of the HAZ (see Figure 3b). As the IWT increased (from 10 °C to room temperature), the small-sized crystalline particles were observed in all areas of the HAZ (see Figure 3c,d). The TEM foils were extracted from the region adjacent to micro-sized crystalline structure by a specific-site FIB technique. Figure 4a shows the bright-field image of the interface between the micro-sized crystalline structure and the BMG matrix, where the micro-sized crystalline structure is composed of a large number of grains with various orientations. In Figure 4b,c, all of the selected area electron diffraction (SAED) patterns, as taken from various regions of the micro-sized crystalline structure, were identified as the Zr_6_CoAl_2_ phase. In addition, several small (hundred nanometers) size crystalline particles, with diameters ranging from 100 to 300 nm, were dispersed in the BMG matrix, and can be observed in the TEM image (see Figure 5a–c). Remarkably, these small crystalline particles appear as the hexagonal prism, indicating a hexagonal lattice structure. The corresponding SAED patterns confirm that the small crystalline particles dispersing in the BMG matrix were the Zr(Al_x_Co_1−x_)_2_ phase with a MgZn_2_ hexagonal Laves structure. The Zr-Co-Al ternary intermetallic compounds, such as Zr_6_CoAl_2_ and Zr(Al_x_Co_1−x_)_2_ phases, were also observed in annealed Zr-Co-Al alloys reported in other literature.

**Figure 6:**
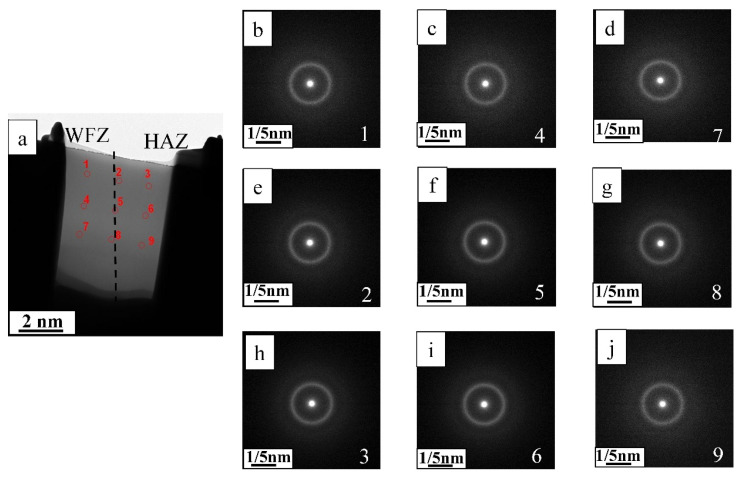
(**a**) BF images of WFZ and HAZ in the surface area of the weld, (**b**–**d**) SAED patterns of the WFZs, (**e**–**g**) SAED patterns at the boundary between WFZ and HAZ, and (**h**–**j**) SAED patterns of the HAZs.

**Figure 7:**
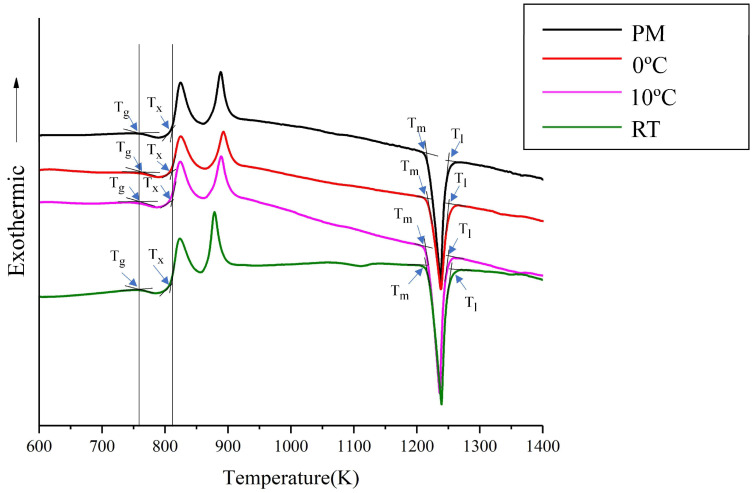
DSC traces measured from the PM and BMG welds with various IWTs.

As described in Section 1, an alloy with the composition of Zr_54_Al_17_Co_29_ can be cast into a rod with a maximum diameter of 6 mm, as reported by Li et al. [1]. However, this BMG alloy system still suffers from poor GFA when compared to Zr/Cu-based (e.g., ZrCuAgAl or ZrCuNiAl) [15,16,17,24] BMGs. Thus, under a low GFA of the alloy, the formation of the crystalline phase, as detected during quenching of the ZrCoAl BMG cast, has seldom been reported by literature [3,4,12,13], though those studies attempted to explore the kinetics of the crystallization behaviors of ZrAlCo. By controlling the time, temperature, and composition ratios, it is possible to obtain composites with a fine crystalline phase dispersed in the glass matrix. Literature has indicated that as GFA decreases (due to the Zr/Co/Al ratio or the addition of a fourth element), the crystalline phases, such as Zr_2_Co, ZrCo, or Zr_2_Al, were detected with a higher Zr/Co ratio. When the Zr/Co ratio and content decreased, the Al content increased and the effect of Al became more obvious, suggesting the dissolution of Al into the Zr/Co phase may occur. Following the addition of the Nb element in the ZrCoAl BMG alloy, the GFA of the alloy was further reduced. Then, after the casting process of the BMGs, the Zr_6_CoAl_2_ phase amount was initially formed in the amorphous matrix of the center alloy due to the lower cooling rate of the area. Following the welding processes, a small (sub-micro or nano-) sized Zr(Al_x_Co_1−x_)_2_ was formed in the HAZ. As the IWT lowered, the volume faction of the small-sized Zr(Al_x_Co_1−x_)_2_ phase was also decreased. While the small-sized crystalline was still observed in the lower IWT of the 0 °C weld, it was only found at 70 μm below the surface of the HAZ. The surface area identified by TEM (see Figure 6) appears to be an amorphous matrix; thus, to investigate the glass transition and crystallization behaviors of the BMG and the BMG welds, DSC (at a heating rate of 20 K/min) was used. Figure 7 shows the DSC traces measured from the PM and BMG welds with various IWTs. All samples showed a glass transition followed by a supercooled liquid region before crystallization. The characteristic temperatures, including glass transition temperature (T_g_), crystallization temperature (T_x_), melting temperature (T_m_), and liquidus temperature (T_l_), as well as the GFA indices, ΔT_x_ (ΔTx = T_x_ − T_g_) and γ_m_ (γ_m_ = (2T_x_ − T_g_)/T_l_) [28], of the BMG and BMG welds are defined and listed in Table 2. As shown in Figure 7 and Table 2, the GFA indicators, ΔT_x_, and γ_m_ of the welds are lower than that of the PM. In addition, the GFA indicators were reduced when the IWTs were increased. Since no crystallization was found in the WFZs of the welds, the reduction in GFA indicators was attributed to the formation of sub-micro or nano-sized Zr(Al_x_Co_1−x_)_2_ in the HAZs of the welds.

**Figure 8:**
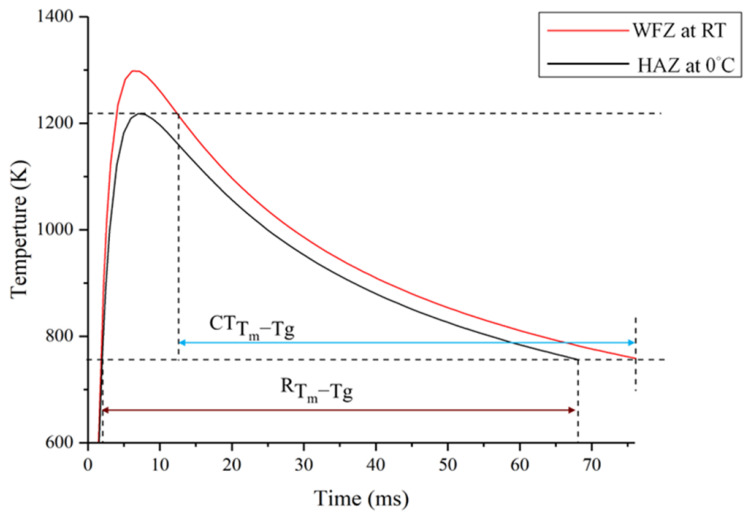
Schematic representation of the crystallization behaviors in the HAZ and WFZ of the Zr-Al-Co-Nb BMG welds.

Furthermore, the study [11] showed that when Ta was added to the ZrCoAl BMG alloy, the PM and the HAZ were crystalline-free when an IWT of 0 °C was applied in laser welding. This may be attributable to the higher GFA (ΔT_x_ = 52 K) [1,11] index of the Zr-Al-Co-Ta ((Zr_53_Al_17_Co_29_) Ta_1_) BMG alloy, as compared to (ΔT_x_ = 48 K) of the Zr-Al-Co-Nb ((Zr_53_Al_17_Co_29_) Nb_1_), which is more likely to be crystallize-free in the PM and the HAZ. Furthermore, no obvious crystallization was observed in the WFZs of the welded samples. Many studies [11,20,29,30,31] have indicated that the crystallization mechanisms in HAZ and WFZ are different. Figure 8 shows the schematic representation of the crystallization behaviors in the HAZ and WFZ of the Zr-Al-Co-Nb BMG welds, which were derived from the refined 2D Rosenthal’s analytical solution [29], as described by Ashby and Easterling [29]. Furthermore, the detailed equations and thermal properties, such as thermal conductivity, specific heat, and density, were used for the analytical solution, as reported in [12].

A solid state reaction of the crystallization was formed in the HAZ and it is highly related to the heating and cooling time (R_Tm/Tg_) [11] of the welding thermal cycle in the crystallization temperature range (i.e., between T_m_ and T_g_), as indicated in Figure 8. However, the peak temperature of the welding thermal cycle in the WFZ is higher than the liquidus temperature. Hence, crystallization in this zone is greatly related to the effective cooling time interval (CT_Tm/Tg_) [28] between T_m_ and T_g_ during solidification. Table 3 indicates the calculated (R_Tm/Tg_) and (CT_Tm/Tg_) values issued from the different IWTs. The simulation results show that R_Tm/Tg_ decreased with the lowering IWTs. The selected parameters and IWTs used in this investigation did cause crystallines in the HAZs of all the welds. From the above microstructural observation, to achieve a crystallization-free HAZ surface, designates parameters of laser welding and an IWT at 0 °C should be used, which had an R_Tm/Tg_ of 64 ms. However, no crystallization was observed in the WFZs of the welds with different IWTs (even the welding samples at room temperature), which can be involved in the lower magnitudes of CT_Tm/Tg_ in the WFZ with various IWTs, as opposed to that of R_Tm/Tg_ in the HAZ with an IWT of 0 °C.

**Figure 9:**
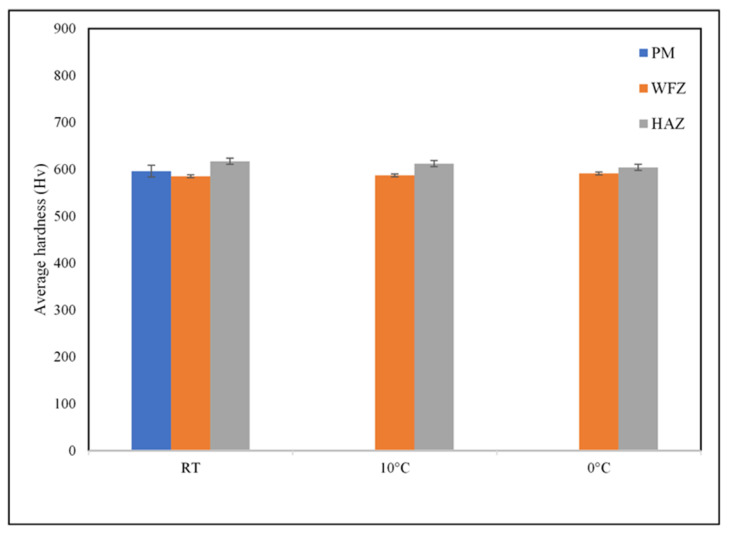
Microhardness test values of the PM, HAZs and WFZs of the laser spot-welded samples under various IWTs.

Vicker’s microhardness testing was conducted to study the mechanical properties in specific areas (PM, HAZ, and WFZ) of the welds. The microhardness test values of the PM, HAZs, and WFZs of the laser spot-welded samples under various IWTs are shown in Figure 9. It was observed that the hardness values of the HAZs in the welded samples are all slightly higher than those of the PM, which may be attributed to the presence of the sub-micro or nano-size of the Zr(Al_x_Co_1−x_)_2_ phases. As described in Section 1, in this study, Nb element was added into the Zr–Al–Co system to improve its toughness and ductility, which led to a softer PM matrix. Therefore, when the sub-micro or nano-sized Zr(Al_x_Co_1−x_)_2_ phases formed in the HAZ, the HAZ samples had higher hardness values. However, as no crystallization was found in the WFZs of the welds with various IWTs, the hardness values of the WFZs of the welded samples are similar to that of the PM.

**Figure 10:**
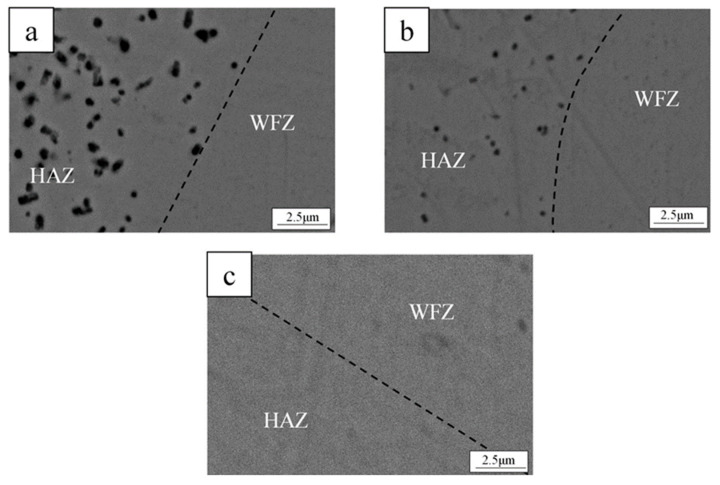
Surface observations of the HAZs and WFZs with the IWTs of (**a**) RT (**b**) 10 °C (**c**) 0 °C after 120 h soaking corrosion test.

To investigate the effects of IWTs on corrosion resistance, the welded samples were soaked in a 6M hydrochloric acid (HCl) solution at 35 °C for various periods (from 72 h to 120 h). After the corrosion testing, pitting conditions were observed by SEM in the PM, HAZ, and WFZ surfaces. No obvious pitting was found after 72 h soaking; after 120 h soaking, pitting could be observed (see Figure 10) on the HAZ surfaces of the welds at the IWTs of RT and 10 °C. At the same time, no obvious pitting was found on the HAZ surface of the weld at the IWT of 0 °C. The size and density of the pits in the HAZ increased with the rising IWTs. Regarding the WFZs, no pitting was observed in any of the welded samples.

Earlier studies [28] indicated that dissolution of the noble elements (such as Nb) in an amorphous matrix can enhance pitting corrosion resistance. For a Zr-Al-Co BMG with added Nb, a passive oxide film [19] may form a barrier layer on the surface. When Zr-Al-Co-Nb BMG came in contact with an aqueous solution containing halide ions Cl^−^, corrosion might be initiated at weak points on the passive film, such as the interface between the crystalline and glass matrix in the HAZ, which opened these sites for Cl^−^ absorption and attack. After the interface was corroded, due to the potential difference between the crystalline and glassy matrix, more severe pit corrosion might occur. In particular, when the IWT was increased, the size and density of the crystalline also increased, and the corrosion pits on the HAZ surface became more severe. However, no obvious pitting was found on the HAZ surface of the weld at the IWT of 0 °C, which might be attributed to the crystalline-free surface of the HAZ. The small size crystalline effect was only observed at the depth of about 70 μm below the surface of the BMG cast plate. Furthermore, after the laser spot welding process, all test samples consisted of an amorphous matrix in all WFZs, which is similar to the PM, and provides excellent corrosion resistance in this area.

Based on the Zr_55_Al_20−x_Co_25_ alloy system with high glass-forming ability (GFA), quaternary Zr_55_Al_20−x_Co_25_Nb_x_ (x = 2.5 and 5 at.%) metallic glasses have been developed. The addition of Nb was found to enhance their resistance to pitting corrosion in a 3 mass% NaCl solution [21]. Furthermore, Zr_53_Al_17_Co_29_Nb_1_ bulk metallic glasses (BMGs) also exhibited improved pitting corrosion resistance with the addition of 1 wt.% Nb in a 6 M HCl solution.

## 4. Conclusions

To investigate the weldability of (Zr53Al17Co29)Nb_1_ BMG, laser spot welding was performed using different initial welding temperatures (IWTs), including room temperature, 10 °C, and 0 °C. The microhardness, thermal properties, and corrosion resistance of the welds were subsequently evaluated, and the main findings are summarized as follows:

1. Due to the lower GFA, a small amount of the Zr_6_CoAl_2_ phase was found in the amorphous matrix of the center area of the BMG cast plate.
2. After the laser welding, a small size Zr(Al_x_Co_1−x_)_2_ was formed in the HAZs of all the welded samples, which produced higher values of microhardness in the HAZ and lower GFA indicator values in the welds.
3. Regarding the lower IWT of the 0 °C weld, the small size HAZ crystalline was observed only at 70 μm below the surface of the HAZ. The corrosion tests on the welds showed better corrosion resistance with lower IWT, as compared to higher IWTs (room temperature or 10 °C).

## Figures and Tables

**Table 1:** The initial parameters for the laser spot welding.

	Laser Voltage (V)	Peak Power (kW)	Pulse Frequency (Hz)	Pulse Duration (ms)	Laser Energy (J)	Spot Size (mm)	Gas Flow Rate (per. min)
A	238	1.3	2.0	4.6	6.0	0.4	10
B	243	1.4	2.0	4.6	6.5	0.4	10
C	249	1.5	2.0	4.6	7.0	0.4	10

**Table 2:** The characteristic temperatures and GFA indices of the BMG and BMG welds.

IWT	$T_{g}$ (K)	$T_{x}$ (K)	$T_{m}$ (K)	$T_{l}$ (K)	$\Delta T_{x}$ (K)	$\gamma_{m}$
PM	758	806	1216	1245	48	0.692
0 °C	758	804	1216	1245	46	0.690
10 °C	759	804	1212	1243	47	0.689
RT	758	802	1212	1246	44	0.686

**Table 3:** Calculated (R_Tm/Tg_) values are derived from the different initial welding temperatures. Here, (CT_Tm/Tg_) value is derived from RT welding.

	RT	10 °C	0 °C
${\mathrm{CT}}_{Tm-Tg}$ (ms) (WFZ)	62	59	56
$R_{Tm-Tg}$ (ms) (HAZ)	72	68	64

## Data Availability

The original contributions presented in this study are included in the article. Further inquiries can be directed to the corresponding author.
